# Supplementary material for: Initiating and imaging the coherent surface dynamics of charge carriers in real space
Source: Nat Commun. 2016 Sep 28;7:12839. doi: 10.1038/ncomms12839 (PMC5052722; doi:10.1038/ncomms12839)
Supplement: Supplementary Information — Supplementary Figures 1-10 [file ncomms12839-s1.pdf]

SUPPLEMENTARY MATERIALS:

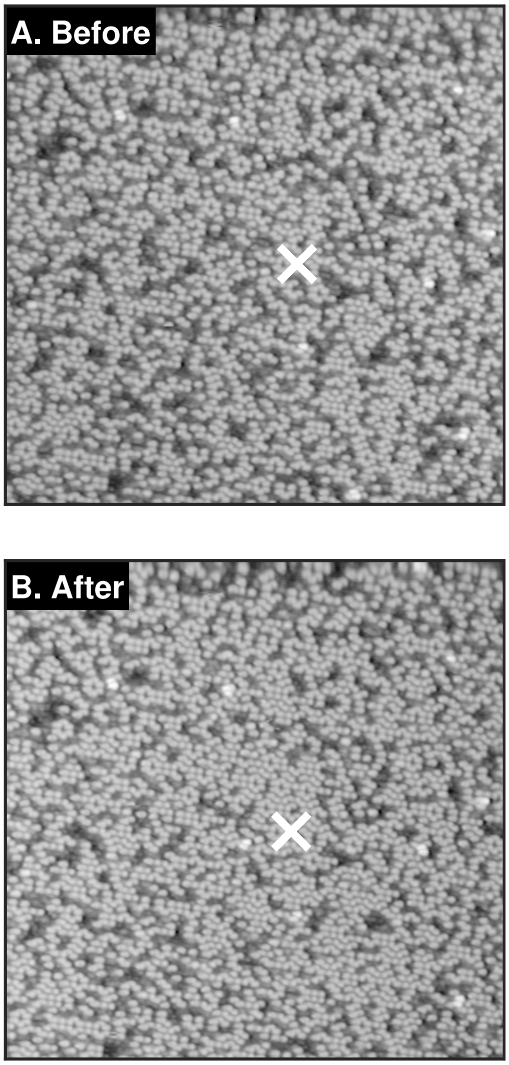

Supplementary Figure 1. Nonlocal manipulation of toluene molecules bonded to unfaulted-middle adatom sites on the Si(111)-7x7 surface at room temperature. STM images (50 nm x 50 nm, +1 V, 100 pA) of toluene on Si(111)-7x7 taken before (A) and after (B) an injection of charge (injection at −1.6 V , 900 pA and 45 s) at unfaulted middle adatom site ‘X. These are larger area images corresponding to Figs. 1A and D.

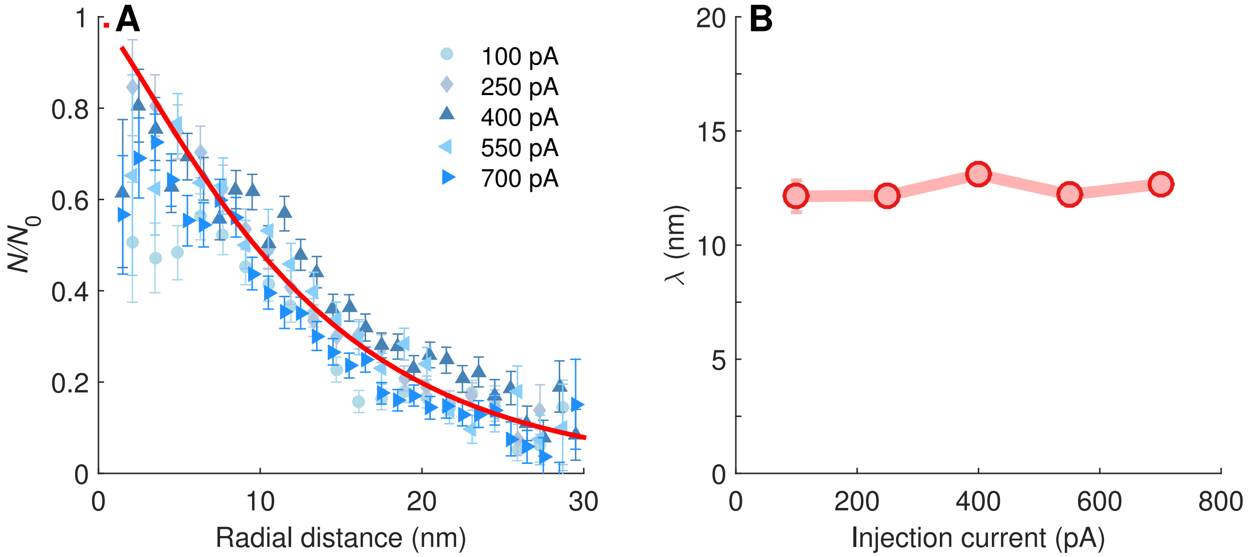

Supplementary Figure 2. Current dependence of nonlocal manipulation of toluene molecules. (A) Radial distributions for the fraction of (all) molecules that are manipulated as a function of radial distance from the injection site. Experiments conducted with constant injection voltage −1.6 V and set total amount of injected charge, but varying the time and the current of the pulse (high current short time, low current long time): 100 pA, 50 s; 250 pA, 20 s; 400 pA, 12.5 s; 550 pA, 9.1 s; and 700 pA, 7.1 s. All radial curves lie on the same line. Error bars are the standard error of 5 injection experiments at each injection current. (B) The values for the diffusion length λ for eq. 1 fitted to curves of A. Since the model of eq. 1, relies on a 1-hole process this invariant λ confirms that the manipulation process is a one hole process. Error bars correspond to one standard deviation.

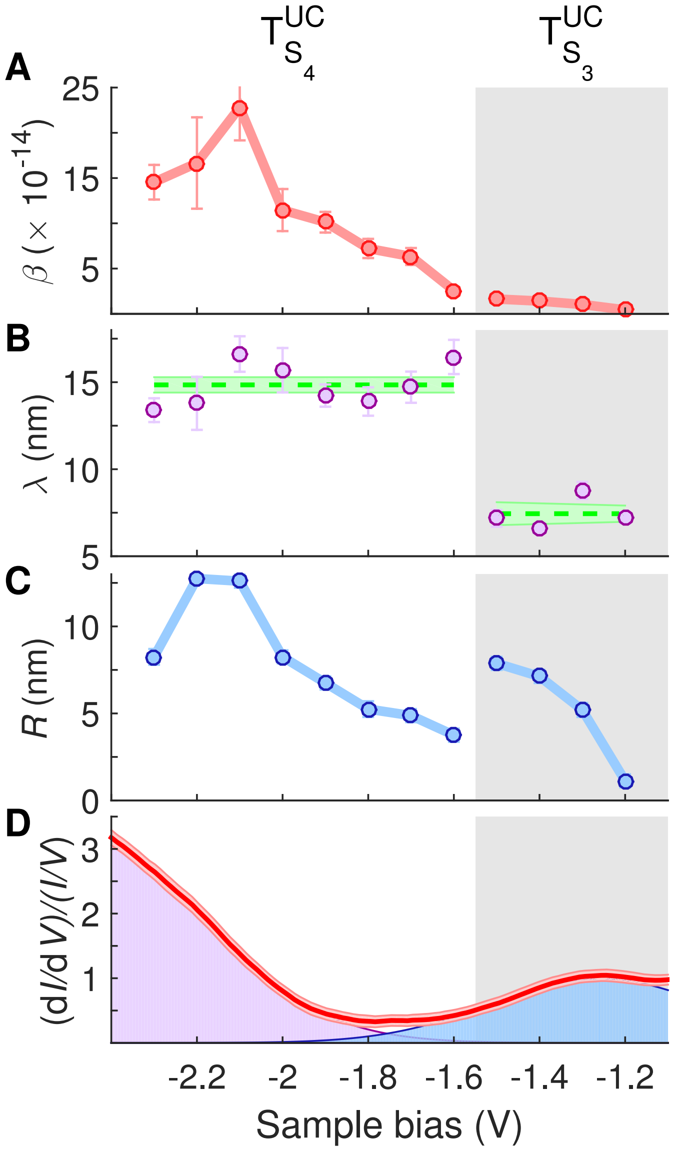

Supplementary Figure 3. UC Molecules: Injection bias dependence for injection into UM adatoms site and nonlocal manipulation of UC toluene molecules. (A) β the probability of manipulation per injected hole, (B) the length-scale λ and (C) the width of the suppression region R. The first transport regime has been shaded to guide the eye. Horizontal green lines in (B) show the average length-scale λ for each transport region with standard error indicated by width of the bar. (D) Variable gap STS of clean UC adatoms. The standard error (just visible) of 38 individual spectra has been shaded. Two Gaussian functions have been fitted as indicated: peak position and FWHM (-1.3 ± 0.6) eV and (-2.5 ± 0.7) eV. All errors quoted are one standard deviation.

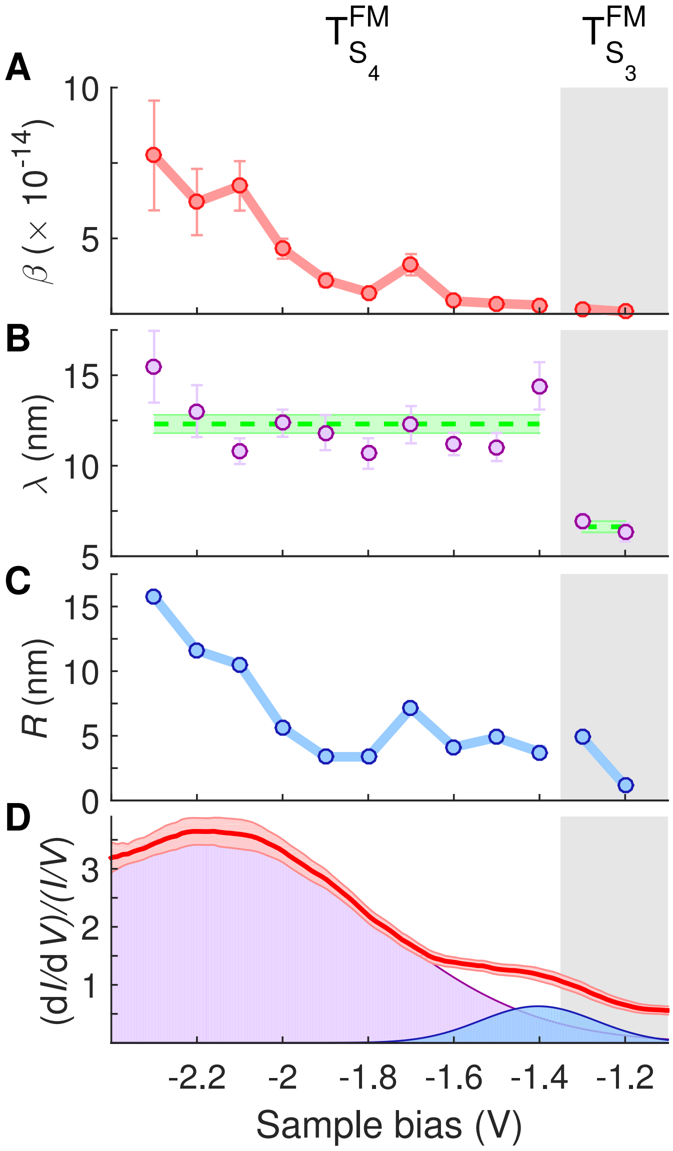

Supplementary Figure 4. FM Molecules: Injection bias dependence for injection into UM adatoms site and nonlocal manipulation of FM toluene molecules. (A) β the probability of manipulation per injected hole, (B) the length-scale λ and (C) the width of the suppression region R. The first transport regime has been shaded to guide the eye. Horizontal green lines in (B) show the average length-scale λ for each transport region with standard error indicated by width of the bar. (D) Variable gap STS of clean FM adatoms. The standard error (just visible) of 38 individual spectra has been shaded. Two Gaussian functions have been fitted as indicated: peak position and FWHM (-1.4 ± 0.3) eV and (-2.2 ± 0.9) eV. All errors quoted are one standard deviation.

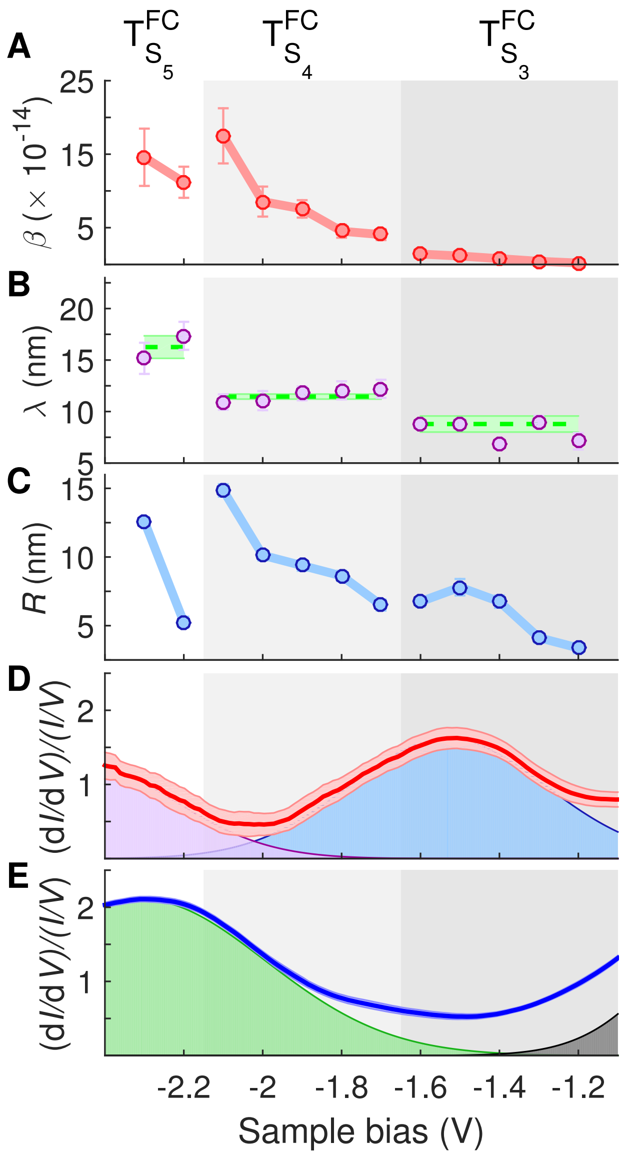

Supplementary Figure 5. FC Molecules: Injection bias dependence for injection into UM adatoms site and nonlocal manipulation of FC toluene molecules. (A) β the probability of manipulation per injected hole, (B) the length-scale λ and (C) the width of the suppression region R. The first transport regime has been shaded to guide the eye. Horizontal green lines in (B) show the average length-scale λ for each transport region with standard error indicated by width of the bar. (D,E) Variable gap STS of clean FC adatoms. The standard error (just visible) of 38 individual spectra has been shaded. Two Gaussian functions have been fitted as indicated: peak position and FWHM: (D) FC adatom site site (-1.5 ± 0.6) eV and (-2.4 ± 0.5) eV. (E) FR (faulted restatom) site (-2.3 ± 0.7) eV. All errors quoted are one standard deviation.

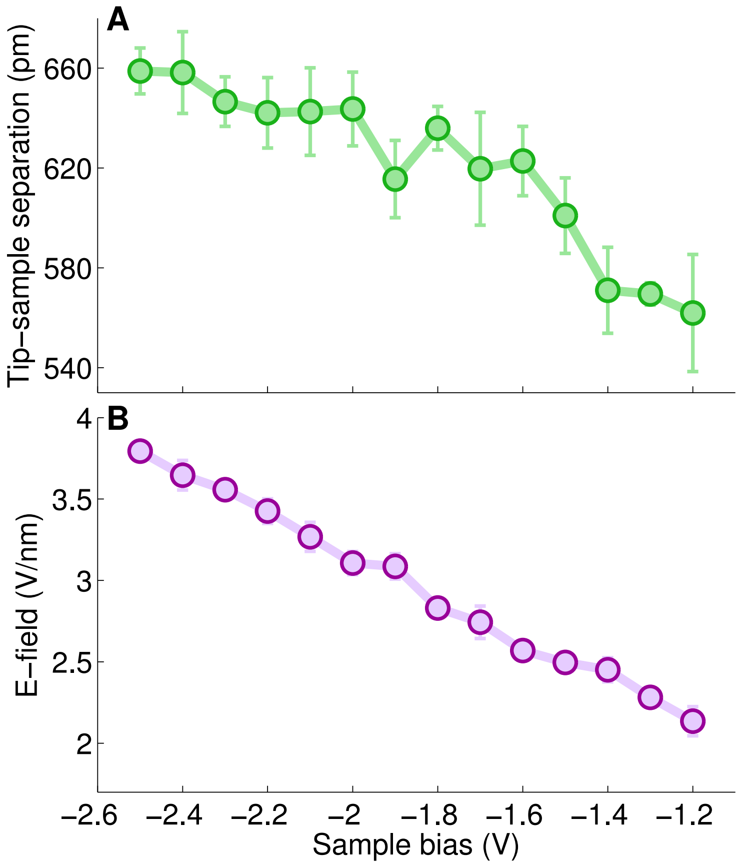

Supplementary Figure 6. Absolute tip height and electric field strength during nonlocal manipulation as a function of injection bias voltage. (A) Absolute tip height above UM site during 900 pA injection. We assume a tip height of 0.7 nm at our set point of +1 V and 100 pA. (B) Magnitude of E-field in the gap calculated from $E = V/z$ where V is the injection voltage and z is the absolute height given in (A). Error bars are the standard error of 5 measurements of the tip-sample separation at each injection bias voltage.

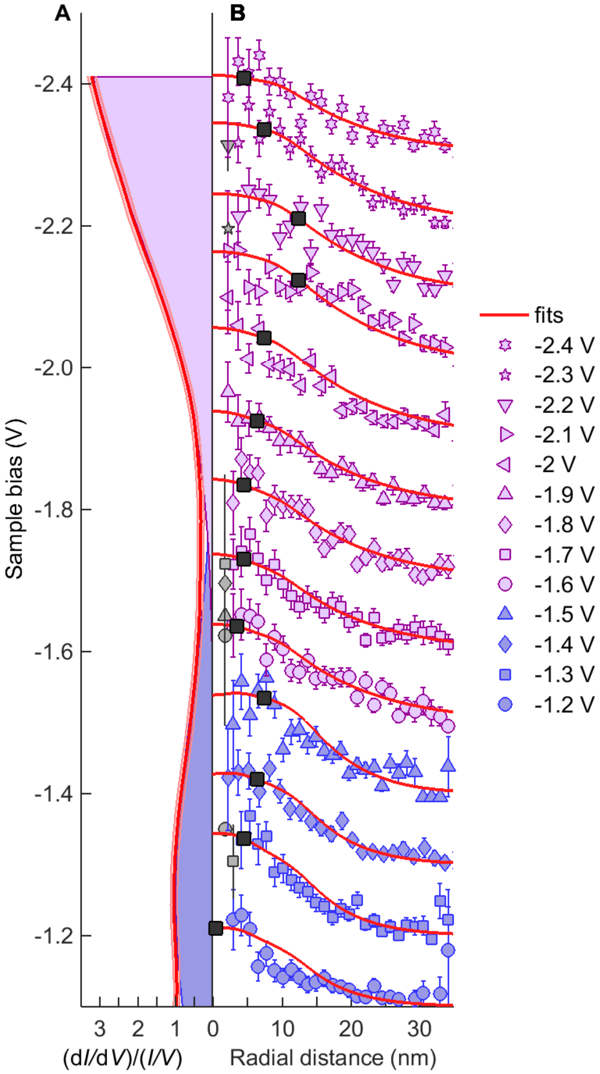

Supplementary Figure 7. UC Molecules: Comparison of STS with nonlocal manipulation and displaced 2D diffusion model. (A) Normalized STS spectrum taken on a clean UC site. Shaded areas show Gaussian fits to the two peaks: peak position and FWHM (-1.3 ± 0.6) eV and (-2.5 ± 0.7) eV. (B) Injection voltage dependence of nonlocal manipulation of UC toluene molecules with a UM injection site. Radial distribution curved have been vertically offset to aid clarity and match STS energy axis. Solid red lines show the inflation model fit to each dataset. Global fits of the inflation-diffusion model give inflation times for $\mathrm{T}_{\mathrm{S}_3}^{\mathrm{UC}}$ $\tau_i = (14 \pm 1)$ fs and $\mathrm{T}_{\mathrm{S}_4}^{\mathrm{UC}}$ $\tau_i = (4 \pm 1)$ fs. Black markers indicate the range of inflation region determined form the experimental data (as given in Supplementary Fig. 3C). Error bars are the standard error of 5 injection experiments at each injection bias voltage.

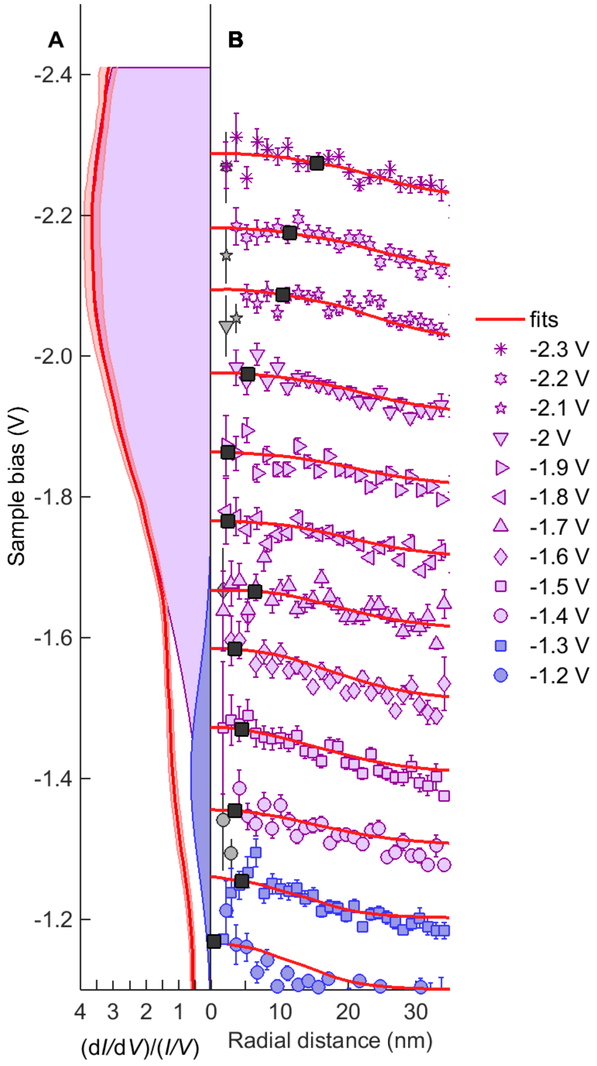

Supplementary Figure 8. FM Molecules: Comparison of STS with nonlocal manipulation and displaced 2D diffusion model. (A) Normalized STS spectrum taken on a clean FM site. Shaded areas show Gaussian fits to the two peaks: peak position and FWHM (-1.4 ± 0.3) eV and (-2.2 ± 0.9) eV. (B) Injection voltage dependence of nonlocal manipulation of FM toluene molecules with a UM injection site. Radial distribution curved have been vertically offset to aid clarity and match STS energy axis. Solid red lines show the inflation model fit to each dataset. Global fits of the inflation-diffusion model give inflation times for $\mathrm{T}_{\mathrm{S}_3}^{\mathrm{FM}}$ $\tau_i = (11 \pm 1)$ fs and $\mathrm{T}_{\mathrm{S}_4}^{\mathrm{FM}}$ $\tau_i = (12 \pm 1)$ fs. Black markers indicate the range of inflation region determined form the experimental data (as given in Supplementary Fig. 4C). Error bars are the standard error of 5 injection experiments at each injection bias voltage.

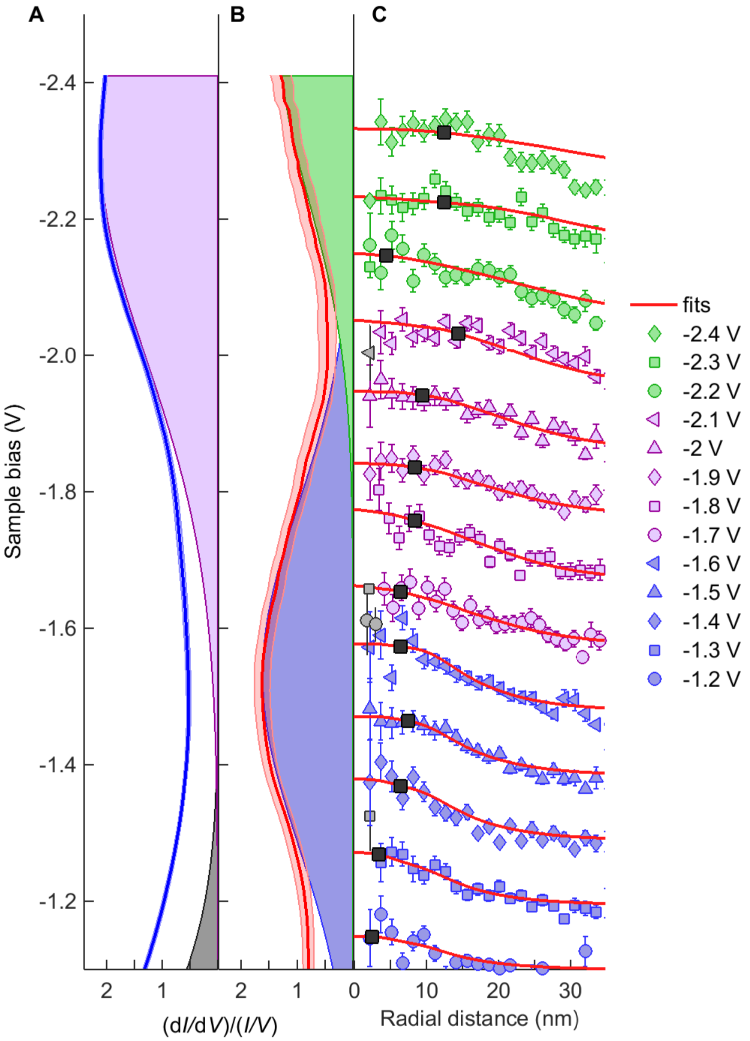

Supplementary Figure 9. FC Molecules: Comparison of STS with nonlocal manipulation and displaced 2D diffusion model. (A) Normalized STS spectrum taken on a clean faulted restatom site, (B) Normalized STS spectrum taken on a clean FC site. Shaded areas show Gaussian fits to the two adatom peaks: peak position and FWHM (-1.5 ± 0.6) eV and (-2.4 ± 0.5) eV and one to the faulted restatom (FR) (-2.3 ± 0.7) eV. (B) Injection voltage dependence of nonlocal manipulation of FC toluene molecules with a UM injection site. Radial distribution curved have been vertically offset to aid clarity and match STS energy axis. Solid red lines show the inflation model fit to each dataset. Global fits of the inflation-diffusion model give inflation times for $\mathrm{T}_{\mathrm{S}_3}^{\mathrm{FC}}$ $\tau_i = (9 \pm 1)$ fs, $\mathrm{T}_{\mathrm{S}_4}^{\mathrm{FC}}$ $\tau_i = (7 \pm 1)$ fs and $\mathrm{T}_{\mathrm{S}_5}^{\mathrm{FC}}$ $\tau_i = (34 \pm 3)$ fs. Black markers indicate the range of inflation region determined form the experimental data (as given in Supplementary Fig. 5C). Error bars are the standard error of 5 injection experiments at each injection bias voltage.

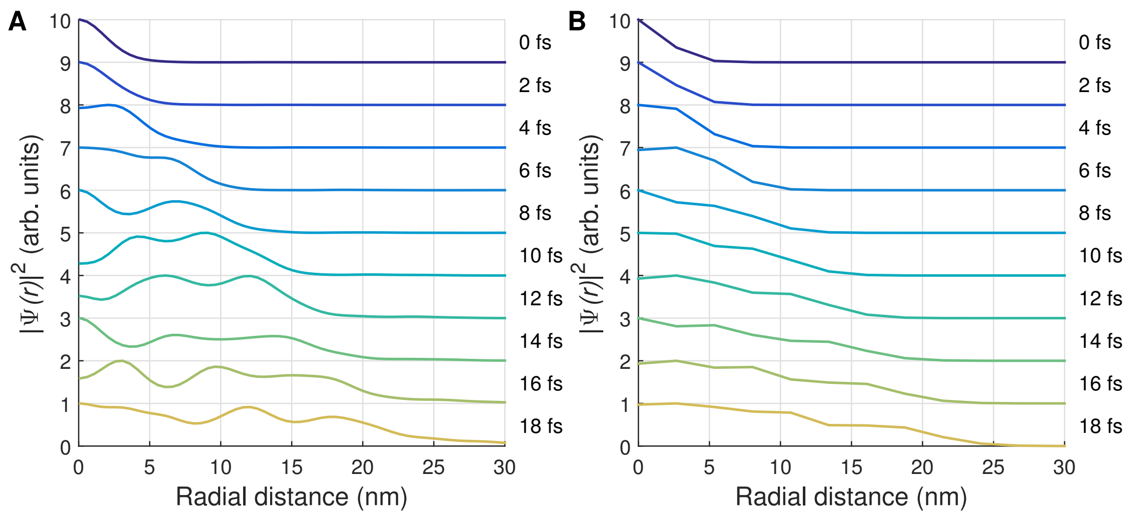

Supplementary Figure 10. Comparison of wavepacket dynamics for (A) free electron like Bessel function eigen-states (as present in Fig. 3) and (B) solving the time-dependent Schrödinger equation assuming the tight binding Hamiltonian on a a hexagonal lattice. See methods section for details. Each shows the radial dependence $|\psi(r)|^2$ (the probability per unit area) from the initial initiation time at $t = 0$ up to an 'inflation' time of 18 fs. Each curve is normalized to its maximum value and is shifted for clarity.
